# All together now: aggregating multiple records to develop a person-based dataset to integrate and enhance infectious disease surveillance in Ontario, Canada

**DOI:** 10.17269/s41997-020-00295-5

**Published:** 2020-02-24

**Authors:** Michael Whelan, Christina Renda, Karin Hohenadel, Sarah Buchan, Michelle Murti

**Affiliations:** 1 Communicable Diseases, Emergency Preparedness and Response, Public Health Ontario, 661 University Avenue, 17th Floor, Toronto, ON M5G 1M1 Canada; 2 Dalla Lana School of Public Health, University of Toronto, Health Sciences Building 155 College Street, 6th Floor, Toronto, ON M5T 3M7 Canada

**Keywords:** Integrated, Infectious diseases, Surveillance, Public health, Intégré, Maladies infectieuses, Surveillance, Santé publique

## Abstract

**Setting:**

Syndemics occur when two or more health conditions interact to increase morbidity and mortality and are exacerbated by social, economic, environmental, and political factors. Routine provincial surveillance in Ontario assesses and reports on the epidemiology of single infectious diseases separately. Therefore, we aimed to develop a method that allows disease overlaps to be examined routinely as a path to better understanding and addressing syndemics in Ontario.

**Intervention:**

We extracted data for individuals with a record of chlamydia, gonorrhea, infectious syphilis, hepatitis B and C, HIV/AIDS, invasive group A streptococcal disease (iGAS), or tuberculosis in Ontario’s reportable disease database from 1990 to 2018. We transformed the data into a person-based integrated surveillance dataset retaining individuals (clients) with at least one record between 2006 and 2018.

**Outcomes:**

The resulting dataset had 659,136 unique disease records among 470,673 unique clients. Of those clients, 23.1% had multiple disease records with 50 being the most for one client. We described the frequency of disease overlaps; for example, 34.7% of clients with a syphilis record had a gonorrhea record. We quantified known overlaps, finding 1274 clients had gonorrhea, infectious syphilis, and HIV/AIDS records, and potentially emerging overlaps, finding 59 clients had HIV/AIDS, hepatitis C, and iGAS records.

**Implications:**

Our novel person-based integrated surveillance dataset represents a platform for ongoing in-depth assessment of disease overlaps such as the relative timing of disease records. It enables a more client-focused approach, is a step towards improved characterization of syndemics in Ontario, and could inform other jurisdictions interested in adopting similar approaches.

## Résumé

**Contexte:**

Les syndémiques surviennent lorsque deux ou plusieurs affections interagissent de façon à augmenter la morbidité et la mortalité et sont exacerbées par des facteurs sociaux, économiques, environnementaux et politiques. En Ontario, les services provinciaux de surveillance de routine évaluent et communiquent séparément l’épidémiologie de chaque maladie infectieuse. Par conséquent, nous avons voulu élaborer une méthode permettant d’examiner de façon routinière les chevauchements de maladies comme moyen de mieux comprendre et traiter les syndémiques en Ontario.

**Intervention:**

Nous avons extrait de la base de données ontarienne sur les maladies à déclaration obligatoire des données concernant des personnes dont le dossier mentionnait la chlamydia, la gonorrhée, la syphilis infectieuse, l’hépatite B et C, le VIH/sida, la maladie invasive à streptocoque du groupe A (SGA) ou la tuberculose de 1990 à 2018. Nous avons transformé les données en un ensemble de données de surveillance intégrée axé sur les personnes (les clients), en retenant celles ayant eu au moins un dossier de 2006 à 2018.

**Résultats:**

L’ensemble de données résultant comprenait 659 136 dossiers de maladie distincts concernant 470 673 clients. Parmi ces clients, 23,1 % avaient plusieurs dossiers de maladie, 50 étant le plus grand nombre pour un client. Nous avons décrit la fréquence des chevauchements de maladies : par exemple, 34,7 % des clients dont un dossier mentionnait la syphilis avaient un dossier relatif à la gonorrhée. Nous avons quantifié les chevauchements connus et conclu que 1 274 clients avaient des dossiers mentionnant la gonorrhée, la syphilis infectieuse et le VIH/sida, puis les chevauchements possiblement émergents, ce qui nous a permis de conclure que 59 clients avaient des dossiers relatifs au VIH/sida, à l’hépatite C et à la maladie invasive à SGA.

**Incidences:**

Notre ensemble novateur de données de surveillance intégrée axé sur les personnes représente une plateforme permettant l’évaluation approfondie en continu des chevauchements de maladies, comme la distribution temporelle des dossiers de maladie. Il favorise une approche davantage axée sur le client, constitue un pas vers l’amélioration de la caractérisation des syndémiques en Ontario et pourrait guider d’autres organisations souhaitant adopter des approches similaires.

## Setting

Syndemics, or synergistic epidemics, occur when two or more health conditions interact to increase morbidity and mortality in a population by more than the sum of their individual effects and are exacerbated by social, economic, environmental, and political factors (Singer 2009). The interaction between HIV, tuberculosis (TB), and poverty or marginalization is often used as an example of a syndemic (Singer et al. 2017), where illness may progress more rapidly with disease co-infection (Public Health Agency of Canada 2014) and with health inequities exacerbating negative health outcomes (Khan et al. 2011; Rivest et al. 2014). A recent community outbreak of invasive group A streptococcal (iGAS) disease in London, Ontario, provides a Canadian example of a potential syndemic. Almost half of the iGAS cases in the outbreak were among marginalized populations, specifically in people who use drugs, people who are underhoused, or both. Many of the cases had hepatitis C (HCV) co-infections and a number of them had HCV-HIV co-infections (Dickson et al. 2018).

In Ontario, infectious disease surveillance is routinely conducted by assessing the epidemiology of individual diseases separately. Therefore, the extent to which infectious disease syndemics are present in Ontario is poorly characterized. Outbreak investigations, such as the iGAS outbreak described above, can identify potential syndemics; however, routine surveillance is not often set up to identify, monitor, and then publicly report on intersecting diseases and their related factors. A first step would be to take a more integrated approach and begin reporting on multiple diseases together (Murti et al. 2019). Some jurisdictions in Canada and the United States have made progress towards a more integrated approach to surveillance and reporting (Alberta Health 2015; County of Los Angeles Public Health 2017; Drobnik et al. 2013; Wong et al. 2018). Other jurisdictions may have an integrated approach, but for various reasons do not publicly report findings. Previous provincial analyses in Ontario looking at multiple and co-infections have been limited in scale with respect to years examined and diseases included and have not been incorporated in routine reporting (Bhanich-Supapol and Whelan 2011; Lee et al. 2011; Ontario Agency for Health Protection and Promotion (Public Health Ontario) 2015). To move towards a better understanding and characterization of the presence of syndemics involving infectious diseases in Ontario, we aimed to produce an integrated surveillance dataset platform to support the analysis of multiple disease events in a single individual.

Our initial objectives were twofold and intended to move us towards integrated infectious disease reporting in Ontario. The first was to develop a novel method to generate a reproducible client-based dataset from which to conduct integrated infectious disease surveillance in Ontario. The second was to conduct descriptive analyses of this initial dataset to assess the frequency of multiple disease events in individuals. This was done using a subset of diseases that have common modes of acquisition and transmission or among diseases with known co-morbidity, namely chlamydia, gonorrhea, infectious syphilis, acute and chronic hepatitis B (HBV), HCV, HIV/AIDS, tuberculosis (TB), and iGAS (Dickson et al. 2018; Public Health Agency of Canada 2014; Public Health Agency of Canada 2019).

## Intervention

In Ontario, “Diseases of Public Health Significance” are required to be reported when identified to local public health units (PHUs) under the *Health Protection and Promotion Act* (Reports, RRO 1990*, Reg 569*). PHUs are also required to conduct case follow-up and management and enter relevant case data into the integrated Public Health Information System (iPHIS). In iPHIS, a person is represented by a client record. iPHIS clients may have many disease records. These may constitute multiple records of the same disease representing separate infections, records of more than one disease type, or both of these.

To complete our first objective, we chose a study population consisting of all iPHIS clients with one or more records of any of the eight diseases of public health significance listed above, occurring between January 1, 2006 and July 15, 2018 (the most recent date of data availability at the time of extraction). To develop our dataset-generating algorithm, we extracted all disease records reported as confirmed cases of the eight diseases that occurred between January 1, 1990 and July 15, 2018. As iPHIS was introduced in 2006, imported client and disease records from the previous information system (January 1, 1990 to December 31, 2005) that were not subsequently cleaned during a new disease record entry in or after 2006 may represent unresolved duplicate client or disease records or records lacking data completeness and quality. Therefore, historical disease records (1990–2005) were only included if associated with clients in the study population.

We transformed all included disease records into a client-based dataset using the unique iPHIS client identifier that is common across all disease records for an individual person and an algorithm developed a priori as described below. Based on iPHIS entry guidelines, clients should not have multiple records of acute HBV, chronic HBV, HCV, and HIV/AIDS. Therefore, if an iPHIS client had multiple records logged for these diseases, we only included the earliest disease record for each of these four disease entities. From 2007 onwards, routine TB genotyping was in place, allowing PHUs to distinguish between new and ongoing TB episodes when two diagnoses occurred within 12 months of each other. Therefore, we included all TB records with a diagnosis in or after 2007. For TB records with a diagnosis prior to 2007, if two or more TB records were found within 12 months of each other, we only included the earliest record. PHUs are directed to ensure that each new disease record entry for chlamydia, gonorrhea, infectious syphilis, and iGAS represents a new infection (versus continuation of a previous infection); therefore, all disease records for these diseases were included. Infectious syphilis, hereafter referred to as syphilis, includes cases of primary, secondary, and early latent syphilis and infectious neurosyphilis.

We analyzed the final dataset to verify algorithm application and to ensure fidelity between original data extract and final dataset.

To complete our second objective, we (1) quantified the total number of clients and disease records; (2) quantified the number of disease records per client; (3) analyzed the number of records per client by disease type; and (4) examined the frequency of multiple record combinations for clients with selected disease types that have common epidemiology or known syndemic potential, specifically the following: (a) HIV/AIDS and TB; (b) HCV, HIV/AIDS, and iGAS; and (c) gonorrhea, HIV/AIDS, and syphilis. Analyses were performed using SAS Enterprise Guide v.7.1 (SAS Institute Inc., Cary, NC).

This project was approved by the Public Health Ontario (PHO) Ethics Review Board and the project team completed a privacy impact assessment (PIA), reviewed by PHO’s Privacy Office. The PIA documents the privacy safeguards for the project, including limiting the use of client identifiers in the Syndemics dataset.

## Outcomes

### Number of client and disease records

**Fig. 1:**
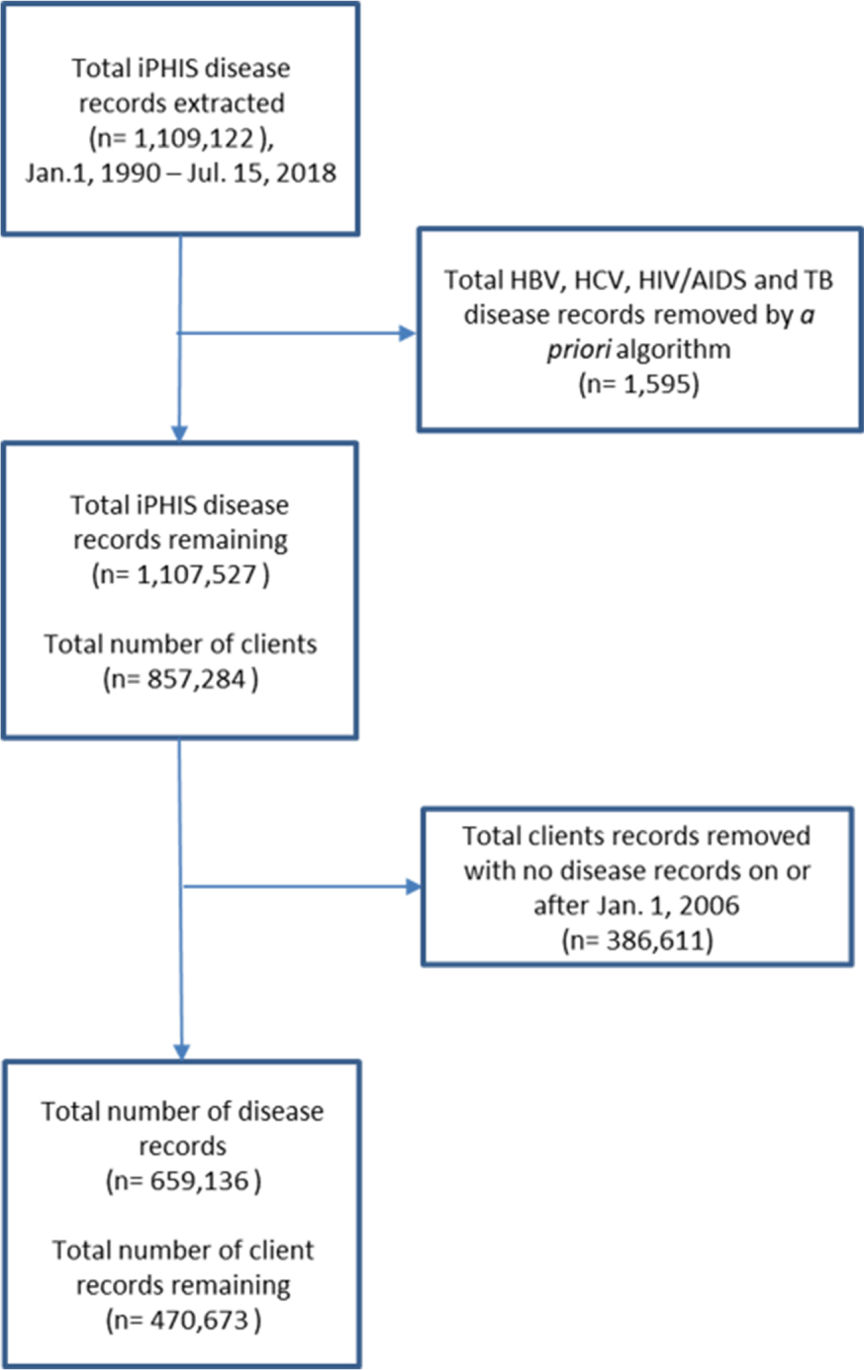
Flowchart illustrating client and disease record inclusion

We extracted 1,109,122 records for our diseases of interest from iPHIS from January 1, 1990 to July 15, 2018. After applying the algorithm for disease record inclusion, 1,107,527 disease records remained, representing 857,284 clients. We removed clients without any disease records in or after 2006, leaving 470,673 clients, representing 659,136 disease records in our final client-based dataset (Fig. 1).

### Number of disease records per client

Of the 470,673 clients in the dataset, the majority (76.9%, *n* = 362,123) had only one disease record (Table 1). While clients with two or more disease records represented 23.1% of all clients, they accounted for 45.1% of disease records. Clients with four or more disease records accounted for 14.1% of disease records despite making up only 3.7% of clients (Table 1). Of the 470,673 clients in the dataset, 72.5% (*n* = 341,282) had one or more chlamydia record. Of the 108,550 clients with more than one disease record, 91.1% (*n* = 98,938) had at least one chlamydia record. No client had records for all eight included diseases. The maximum number of disease types reported per client record was six, including chlamydia, gonorrhea, HCV and HIV/AIDS, syphilis, and HBV (3 clients), or iGAS (2 clients). The maximum number of disease records per client, including multiple records of the same disease type, was 50.

**Table 1:** Percentage of clients by number of disease records and the percentage of disease records these clients represent

Number of disease records per client	Number of clients	% of all client records	Number of disease records associated with these clients	% of all disease records
1	362,123	76.9	362,123	54.9
2	70,041	14.9	140,082	21.3
3	21,273	4.5	63,819	9.7
4+	17,236	3.7	93,112	14.1
Total	470,673	100	659,136	100

### Number and type of disease records per client

Across disease type combinations, percentages of clients with at least one disease record for two different disease types ranged from 0.1% to 56.0% (Table 2). Clients with at least one chlamydia record were the least likely to have another disease type (11.6%) while clients with at least one syphilis record were the most likely to have another disease type (63.0%). Chlamydia records were common among clients with other STI records; 56.0% of clients with at least one gonorrhea record and 34.0% of clients with at least one syphilis record had at least one chlamydia record. The presence of at least one gonorrhea (34.7%) or HIV/AIDS (31.6%) disease record was common among clients with at least one syphilis record. Clients with an HIV/AIDS record often had a disease record of another type of STI, 19.9%, 20.1%, and 21.7% for chlamydia, gonorrhea, and syphilis, respectively (Table 2).

**Table 2:** Clients by disease record type and the percentage with record(s) for a different disease by type

Client has at least one disease record for:	Percentage of clients with an additional disease record for:								
	Chlamydia	Gonorrhea	HBV	HCV	HIV/AIDS	iGAS	Syphilis	TB	Any of the other diseases
Chlamydia (no. of clients = 341,282)	N/A	9.2	0.6	1.6	0.7	0.1	0.9	0.1	11.6
Gonorrhea (no. of clients = 55,925)	56.0	N/A	0.9	3.8	4.5	0.2	5.3	0.1	61.2
HBV (no. of clients = 33,189)	6.3	1.4	N/A	3.0	1.5	0.2	0.8	0.9	11.7
HCV (no. of clients = 56,416)	9.6	3.8	1.8	N/A	2.6	1.3	0.8	0.4	16.0
HIV/AIDS (no. of clients = 12,493)	19.9	20.1	4.1	11.6	N/A	0.7	21.7	1.7	47.9
iGAS (no. of clients = 7968)	4.3	1.6	0.7	9.5	1.1	N/A	0.2	0.2	13.6
Syphilis (no. of clients = 8598)	34.0	34.7	3.0	5.6	31.6	0.2	N/A	0.3	63.0
TB (no. of clients = 8100)	3.7	1.0	3.6	2.5	2.6	0.2	0.3	N/A	12.0

### Examining multiple records between different disease types

**Fig. 2:**
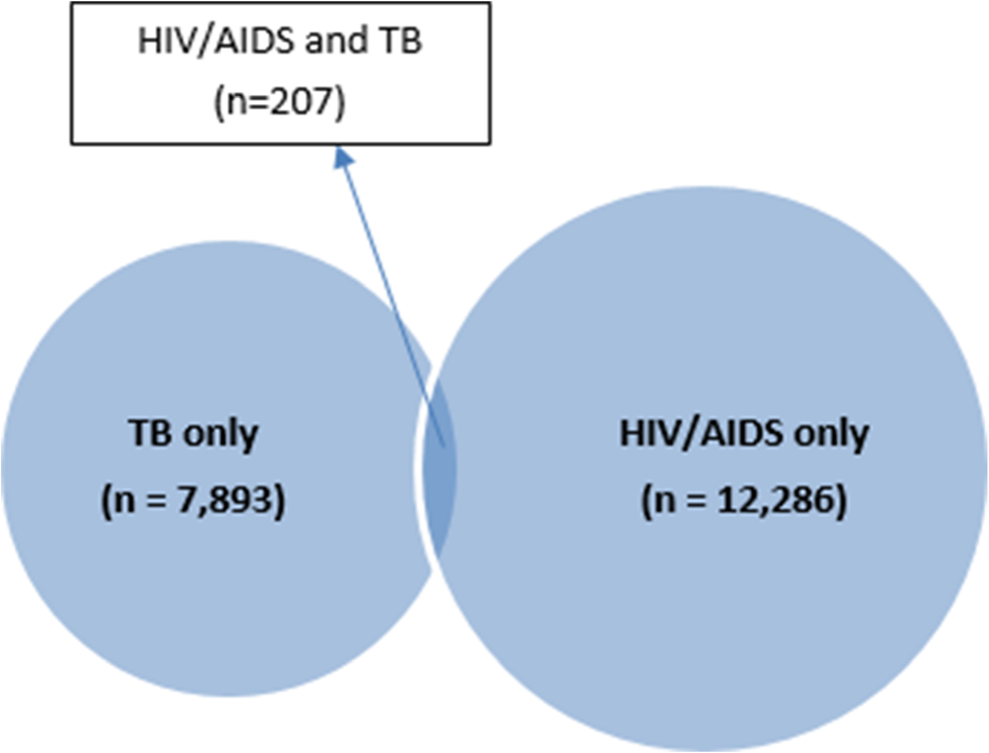
Presence of HIV/AIDS and TB records among clients with at least one record of HIV/AIDS or TB

Of clients with HIV/AIDS or TB records, 1.7% (207/12,493) of clients with an HIV/AIDS record had a TB record and 2.6% (207/8100) of clients with a TB record had an HIV/AIDS record (Fig. 2).

**Fig. 3:**
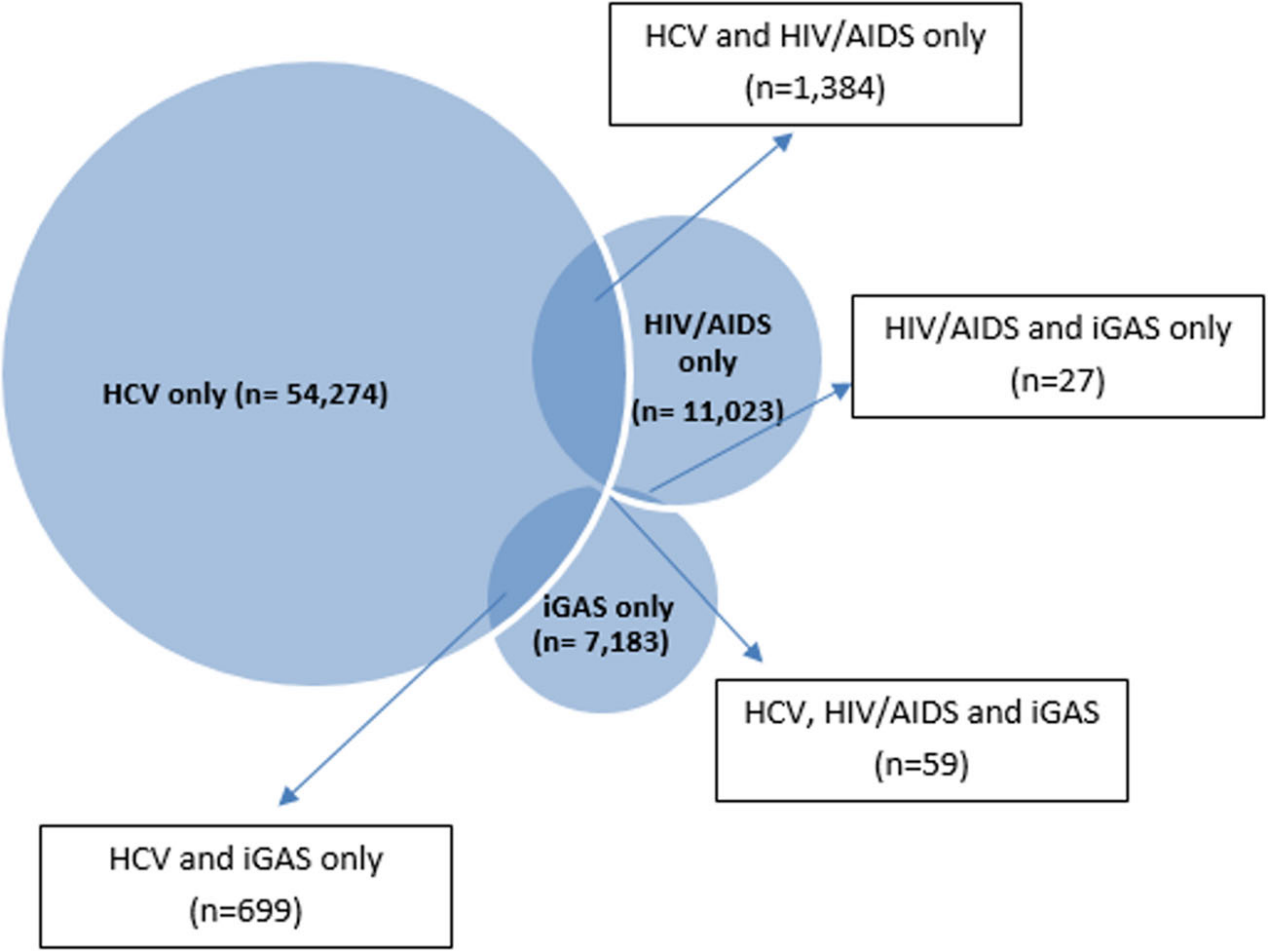
Presence of HCV, HIV/AIDS, and iGAS records among clients with at least one record of HCV, HIV/AIDS, or iGAS

HCV records were present in 11.6% (1443/12,493) of clients with an HIV/AIDS record and 9.5% (758/7968) of clients with an iGAS record (Fig. 3). HIV/AIDS and iGAS were observed in 86 clients, accounting for 0.7% and 1.1% of records among clients with HIV/AIDS and iGAS, respectively. There were 59 clients with records for all three infections, accounting for less than 1.0% of records among clients with any one of the three infections (Fig. 3).

**Fig. 4:**
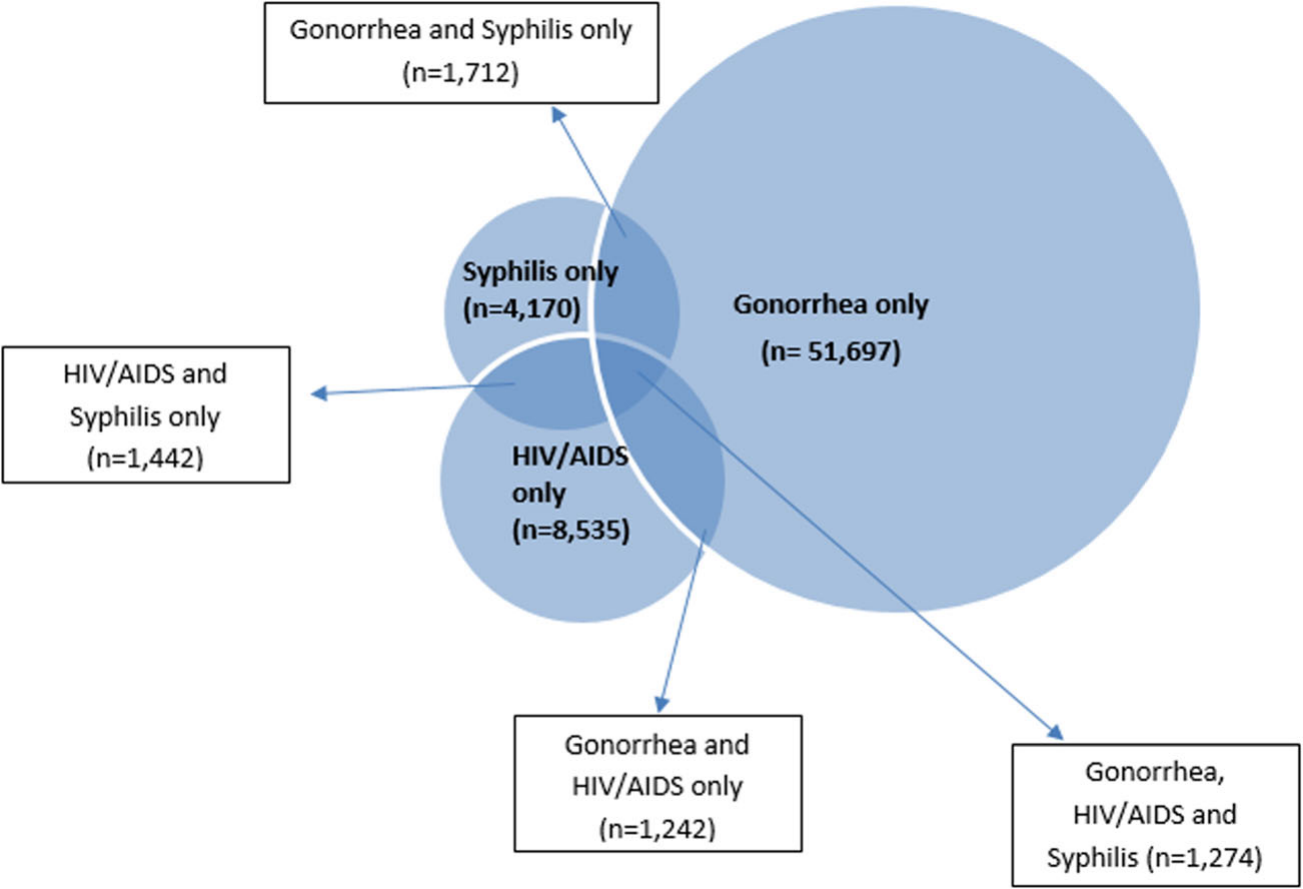
Presence of gonorrhea, HIV/AIDS, and syphilis records among clients with at least one record of gonorrhea, HIV/AIDS, and syphilis

Examining clients with at least one record of gonorrhea, HIV/AIDS, or syphilis, we found 1274 clients with records for all three (Fig. 4). This accounts for 14.8% (1274/8598) of clients with a syphilis record, 10.2% (1274/12,493) of clients with an HIV/AIDS record, and 2.3% (1274/55,925) of clients with a gonorrhea record (Fig. 4).

## Implications

This article describes our process and algorithm for successfully creating a reproducible client-based dataset for quantifying the degree to which individuals in Ontario have multiple records of epidemiologically linked diseases, which is a first for infectious disease surveillance in Ontario. Moreover, our methodology serves as a platform from which a client-based dataset can be produced and updated to support the ongoing analysis of any combination of diseases reported in the province that are of interest for integrated surveillance based on related epidemiology or syndemic potential. The methodology is flexible enough that additional variables collected during routine public health follow-up of cases, such as demographics and risk factors, can be added to the dataset for in-depth analyses.

In addition to overcoming initial technical data manipulation challenges, our implementation was facilitated by three factors. First, under Ontario’s Personal Health Information Protection Act 2004 (Personal Health Information Protection Act 2004), PHO is permitted to collect, use, and disclose data for purposes as described in PHO’s enabling legislation (Ontario Agency for Health Protection and Promotion Act 2007), including surveillance. Second, having an integrated database with common client identifiers facilitated disease record linkage compared with previously described barriers experienced in other jurisdictions with siloed data sources (i.e., HIV data stored separately) (Gasner et al. 2014). Third, analysis from a single provincial database enabled a more comprehensive assessment of the existence of multiple records per client where disease records may be associated with various PHUs in the province. Smaller jurisdictions may be able to repeat these methods to inform local planning, but may not identify the existence of multiple records per client due to small counts. Smaller jurisdictions may also risk missing disease records if there is frequent movement of clients across their boundaries where their disease events are then attributed to other jurisdictions where they do not have access to the records for surveillance purposes.

Beyond establishing the proof-of-concept client-based dataset, our preliminary analyses using the eight included diseases also provided important previously undescribed insights into the intersection of sexually transmitted and bloodborne infections (STBBI) and related infections of TB and iGAS in the province. Interestingly, we found that 23.1% of clients had more than one disease record, representing close to half (45.1%) of all of the disease records. We have not found other reports describing the proportion of clients with more than one disease record of the diseases included in our analysis to compare our findings. In terms of disease types, we found that the occurrence of having at least one other disease type ranged from 12% (chlamydia, HBV, and TB) to 63% (syphilis). These proportions of at least one other disease type are similar to previously published results of an analysis from the New York City Department of Health and Mental Hygiene (NYC DOHMH), where 11% (HBV) to 64% (syphilis) of their client records had more than one disease type. However, their dataset did not include iGAS and required deterministic matching to link records across databases (Drobnik et al. 2014). In both our analysis and the findings from NYC DOHMH, infection with two or more types of diseases was the most common among those with syphilis, followed by gonorrhea (52% in NYC DOHMH) (Drobnik et al. 2014). Further analysis is required to explore these results, particularly with respect to the timing of the syphilis or gonorrhea disease events in relation to other STBBI disease types, and the implications this may have for STI prophylaxis and prevention (Molina et al. 2018; Tan et al. 2017).

We also examined the presence of three specific disease type combinations with known interactions to begin to explore how these methods can be used to support surveillance in Ontario. First, we assessed HIV/AIDS and TB and found 2.6% of TB cases also had an HIV/AIDS record, which is similar to findings in previous analyses of Ontario data (Ontario Agency for Health Protection and Promotion (Public Health Ontario) 2015) and is in line with or lower than the percentage of TB cases with HIV/AIDS co-infection in other jurisdictions (BC Centre for Disease Control 2019; Public Health Agency of Canada 2014; Rivest et al. 2014). Further analysis is required to determine the timing of TB and HIV/AIDS relative to each other, and whether initiatives to routinely screen for HIV/AIDS or TB at the time of TB or HIV/AIDS diagnosis, respectively, have led to changes in timing of identification or prevalence of overlapping infections (Public Health Agency of Canada 2014; Public Health Agency of Canada 2019).

Second, despite the overlap in modes of acquisition/transmission between HIV/AIDS and hepatitis C and iGAS, specifically sharing of drug use equipment, there were only 59 clients in our dataset with all three disease types. Data from the aforementioned London, Ontario, iGAS outbreak investigation indicated that 9.5% of individuals (14/147) with iGAS infections were positive for both HCV and HIV (Dickson et al. 2018). These individuals would represent 23.7% (14/59) of the clients with HCV, HIV/AIDS, and iGAS records in our dataset. This outbreak occurred recently, between April 1, 2016 and February 28, 2018, and therefore, the combination of HIV/AIDS, HCV, and iGAS may be an emerging issue in the province, particularly among people who use injection drugs. Continued and ongoing monitoring of this disease overlap is important to inform prevention and response efforts.

Third, we identified 1274 clients in our dataset with records of HIV/AIDS, gonorrhea, and syphilis, representing over 10% of clients with HIV or syphilis records. While HIV–syphilis co-infection and HIV–gonorrhea co-infection have been previously assessed in separate unpublished analyses, in Ontario, this is the first time records of all three have been examined together at a provincial level. As described above, understanding the overlaps of STBBIs can inform prevention activities. This type of analysis also supports integrated approaches to STBBIs as described in the Pan-Canadian Framework for Action on STBBIs (Public Health Agency of Canada 2018).

### Limitations

While our aim is to develop novel methods of analyzing infectious disease surveillance data, these data have several limitations. As with all passive surveillance systems, only cases of diseases reported to public health and recorded in iPHIS were included in our dataset. Our dataset may be an underrepresentation of the true disease burden in Ontario. This underreporting may vary by disease due to factors such as disease awareness, health-seeking behaviours, availability of health care, severity of illness, clinical practice, methods of laboratory testing, and reporting behaviours. Laboratory testing methods and provincial case definitions for some diseases have changed over time which may also impact the number of cases reported (Ontario Agency for Health Protection and Promotion (Public Health Ontario) 2018). Our dataset may underestimate the number of people with multiple disease records and the number of records per client if an individual was entered in duplicate as two separate clients. Individuals testing positive for HIV anonymously (Government of Ontario 2014) will result in an anonymous client with only the HIV record associated with it and cannot be linked to other disease types. Our dataset may also overestimate the number of records per client, particularly for STIs, where multiple instances of the same infection episode were entered as separate disease records, such as follow-up positive testing results being counted as a new infection. However, this is likely minimized as PHUs assess whether each infection is likely to be the same versus a new infection. Conversely, it is also possible that separate infections occurred and were only entered into iPHIS once.

## Summary

Our initial description of client-based communicable disease surveillance is a step towards better characterization of syndemics in Ontario. While future analyses using additional client and disease record data will allow more fulsome exploration of syndemics, the findings from this initial analysis reveal the degree to which STBBI and epidemiologically related disease events (TB and iGAS) occur and overlap in Ontario. We hope that these findings enable a more client-focused approach to surveillance for Ontario and inform other jurisdictions interested in adopting/adapting similar approaches. While we have identified interesting disease type combinations, we cannot say whether they constitute syndemics without more detailed analyses and socio-demographic information. Therefore, important next steps include assessing the timing between different infections and incorporating additional variables such as age, gender, geographic location, and risk factors. Specific factors of interest could be examined in the future, such as psychoactive drug use among clients with STBBI records or the prevalence of homeless/underhoused and injection drug use among those with BBIs. These additions will help us understand who is at disproportionate risk of having multiple infectious diseases in Ontario and possibly those who may be more likely to transmit those infections, potentially helping to inform more targeted public health interventions. Future considerations also include determining processes for surveillance reporting to public health stakeholders and prioritizing the potential analyses from this platform methodology based on public health needs. Ultimately, the goal of our syndemic-based approach is to inform public health interventions.
